# Geographical Distribution and Socio‐Economic Importance of *Raphia ruwenzorica* From South‐West of Burundi

**DOI:** 10.1002/pei3.70109

**Published:** 2026-01-04

**Authors:** Jacques Nkengurutse, John Maina Nyongesa, Eric Nimubona, Gaëlle Ndayizeye, Anatole Bukuru, Rosette Irampagarikiye, Marie Violette Niyonsaba, Longin Ndayikeza, Kamaguru Bienvenu Imani, Jérémie Ngezahayo, Yuelin Li

**Affiliations:** ^1^ Center of Research in Natural and Environmental Sciences, Department of Biology, Faculty of Sciences University of Burundi Bujumbura Burundi; ^2^ Department of Biological Sciences Jaramogi Oginga Odinga University of Science and Technology Bondo Kenya; ^3^ Center of Research in Natural and Environmental Sciences Doctoral School of University of Burundi Bujumbura Burundi; ^4^ Institut Supérieur de Développement Rural Uvira Democratic Republic of Congo; ^5^ École Doctorale de l'Université du Burundi Bujumbura Burundi; ^6^ Department of Chemistry, Faculty of Sciences University of Burundi Bujumbura Burundi; ^7^ South China National Botanical Garden, University of Chinese Academy of Sciences Guangzhou China

**Keywords:** abundance, altitude, *Raphia ruwenzorica*, socioeconomic

## Abstract

The genus *Raphia* is among the commonly used and socio‐economically important plants in Africa. While *Raphia ruwenzorica* species is known to occur in Burundi, eastern Democratic Republic of Congo, Rwanda, and Uganda, its geographical distribution and socioeconomic importance are poorly documented in Burundi. This study maps *R. ruwenzorica* species from the South‐West region and identifies its socio‐economic importance for the local communities. Different uses and value of the products were investigated using semi‐structured questionnaires on 399 respondents, of which 181 were valid and used for analysis. The number of stands of the species and biophysical properties (diameter and height) were also determined through field inventories. The study site was dominated by adults before flowering compared to the young and adult flowering. The trunks were slender with slightly similar diameter at breast height ranging from 0.42 ± 0.07 to 0.60 ± 0.18 m for all the tree stands. The species distribution ranged from 1300 to 1900 m with predominance between 1600 and 1900 m. *R. ruwenzorica* plant is used as firewood, construction of fences, manufacture of baskets, beehives, and doors, manufacture of brooms, mats, and exhibition clothes of “Intore” and “Umuyebe” dancers (traditional ceremonial costumes). The value of some products ranged between $2.88 and $57.7 US dollars per year. However, this species seems to be threatened by the expansion of agriculture and hence traditional heritage is disappearing. The quasi absence of this species in the low altitudes would be explained by the expansion and the preference of the population for the culture of palm oil. Conservation measures should be enhanced through on‐farm programs such as tree nurseries.

## 1 Introduction

Tropical forest occupies 45% of the total world's landmass and it is the home of approximately 66% of plant species (FAO 2020). The genus *Raphia* is a much conspicuous taxon in these tropical Africa, particularly in humid forests, swamps, and riparian zones. The genus has been reported to decline from its natural habitat due to overexploitation (Donou Hounsode et al. 2016). Palms of the Raphia genus are widely distributed within the lowlands, wet valleys and most often on the riparian sites of rivers and streams as well as on marshy land (Rabarivola 2012). Raphia palm occurs naturally in Africa where it dominates the Guinean forest zone from Cameroon to Angola via Gabon and Congo (Adakaren 2014). This palm is a very important domestic economic resource where all the plant parts are exploited for food, construction of fences and structures for domestic animals (Alladatin 2015; Donou Hounsode et al. 2016; Ouattara et al. 2015; Mogue Kamga et al. 2020; Tata et al. 2023). The sap obtained from Raphia palm is fermented to obtain palm wine (Kamta et al. 2021) and its sweet taste is a characteristic of the amount of sucrose present (Obahiagbon and Osagie 2007; Azuokwu et al. 2024). These African palms also provide other important ecosystem services such as water purification, limiting soil erosion and carbon sequestration (Dargie et al. 2017; Ouattara et al. 2023).

Raphia palm is widely distributed in Africa with most species occurring from west Africa through to eastern and southern Africa (Dransfield et al. 2008). Majority of the recorded species is found in Central Africa with high diversity in Cameroon and Gabon (Couvreur and Sunderland 2019). This distribution is influenced by climate with Raphia adapting well in riparian habitats such as swamps, near streams within the tropical rain forests, gallery forests, and savannas. In addition, Raphia species are found in lowland African regions between 800 and 2000 m in parts of Cameroon, Nigeria and Democratic Republic of the Congo (Mogue Kamga et al. 2020). Twenty‐two palm species of the genus *Raphia* have been identified in Africa by various authors (Helmstetter et al. 2020; Mogue Kamga et al. 2018, 2019; Ouattara et al. 2014; Tagne et al. 2013). Few studies have tried to synonymize the name *R. mambillensis* with *R. vinifera* and provided detailed morphological description of *R. vinifera* based on the study of herbarium material and field data in West Africa (Mogue Kamga et al. 2019). *R. ruwenzorica* is only known from Burundi and eastern of Democratic Republic of Congo and around Lakes Edward and Kivu (Stauffer et al. 2014). Its presence in Rwanda and Uganda has also been reported but no herbarium specimens have been collected (Stauffer et al. 2014). Data from the Global Biodiversity Information Facility show that the species is found in sites located in and around Rwenzori Mountains National Park, Virunga National Park and Kivu Lake in Democratic Republic of Congo. Within Burundi, only one herbarium sample available reveals the species from Mabayi commune in Cibitoke Province near Rwandan border in North‐Western of Burundi (see −2.7 lat, 29.25 long as cited at: https://www.botanicalcollections.be/specimen/BR0000013288679). Our preliminary observations show that the species distribution ranges from North‐Western to South‐West of Burundi in Mumirwa natural region. Mumirwa region represents 10% of the country's total surface area, located in western escarpment of the Lake Tanganyika and Rusizi river plains from 1000 to 1700 m a.s.l. with a mean annual temperature ranging from 18°C to 28°C (Nzigidahera 2012; Havyarimana et al. 2023). The species is common outside forests and protected areas and dominates agricultural land belonging to the community (Helmstetter et al. 2020). However, no specific action for its conservation has yet been undertaken. Due to agricultural expansion and receding river banks (Mphoweh et al. 2015), this plant species is therefore exposed to a potential threat of extinction. At the same time, the significant exploitation of this resource is not accompanied by the expansion of its distribution area and/or its natural regeneration (Donou Hounsode et al. 2016). Gradual loss of the population of *R. ruwenzorica* will continue unless conservation measures are established. Furthermore, knowledge of the species abundance and distribution in Burundi as well as its socioeconomic importance is yet to be unveiled. This study aimed at establishing the geographical distribution, the abundance and socio‐economic importance of *R. ruwenzorica* in Burundi. Except the one herbarium record from Meise Botanic Garden (BR), to the best our knowledge, no‐previous herbarium collection or study have investigated the *R. ruwenzorica* in Burundi.

This study aimed to establish the geographical distribution, abundance, and socio‐economic importance of *R. ruwenzorica* in Burundi. The following research questions guided this study: (1) What is the geographical and altitudinal range of *R. ruwenzorica* in the South‐West of Burundi? (2) What is the population structure of the species in this region? (3) What are the different uses and economic value of *R. ruwenzorica* for local communities? (4) What are the primary threats to the species and the local perception of its conservation status?

## 2 Materials and Methods

### 2.1 Description of the Study Area

This study was carried out in the natural region of Mumirwa in Bujumbura province within the communes of Kanyosha, Nyabiraba, Kabezi, and Mutambu and the communes Buyengero, Burambi, Bugarama, and Muhuta of Rumonge province. This region is located between the Imbo plain (and Lake Tanganyika) and the high peaks of the Congo‐Nile Ridge that forms part of the western slope of the Congo‐Nile Ridge. Previous studies reported that the study area has been dominated by savannah and miombo woodlands as well as forest galleries (Lasserre et al. 1979). Currently, the region is mostly occupied by agricultural fields. This is the heart of flourishing and highly profitable regional palm oil. Growth of palm trees is favored by the altitude and climate of the two provinces. The natural vegetation has quasi‐disappeared except for some relicts of some forest galleries. This is a region characterized by very steep slopes varying from 70% to 100%. The average annual temperature and rainfall vary from 18°C to 28°C and from 1100 to 1900 mm respectively (MEEATU 2013). All the rivers coming from the high mountains as part of the Congo Basin pass through this region. The soils are geologically young, fertile but exposed to very severe erosion with gullies and landslides, and vary according to the topographical conditions (Musonerimana et al. 2025). At the bottom of the valleys, there are deep soils rich in humus while on the steep slopes, there are poor soils washed away by erosion. The region is predominantly inhabited by the Banyaruguru ethnic group, whose livelihoods are primarily based on subsistence farming, palm oil cultivation, and artisanal fishing along Lake Tanganyika. The utilization of Non‐Timber Forest Products (NTFPs) is a vital component of the local economy and cultural practices. While this study focuses on *Raphia ruwenzorica*, other common NTFPs in the region include species such as *Markhamia lutea* and *Syzygium guineense*, used for timber, medicine, and food. The study site has a total population of 429,171 according to the 2008 population census. Inhabitants are mainly farmers and artisans who depend on rice, fish, and palm for their livelihood. This site was selected based on the preliminary surveys and the single known herbarium record indicating the presence of *R. ruwenzorica* in western Burundi. The region's location within the Mumirwa natural region contains the riparian and wetland habitats preferred by the species (Bitama 2023). Additionally, the high human population density in the area allows for a robust assessment of socio‐economic interactions.

### 2.2 Data Collection Methodology

#### 2.2.1 Ecological Data

The collection of ecological and abundance data in the field was carried out from January to April 2021 and July to October 2021 in the natural region of Mumirwa in Bujumbura and Rumonge provinces. As the species is found along the riparian sites of rivers and its tributaries, the inventory method involved following the course of rivers and its tributaries, counting the number of stands (distinct, spatially separated group of *R. ruwenzorica* individuals, with a minimum of 10 m of non‐Raphia vegetation separating it from another group) of the species in three categories: (i) young stands (individuals whose leaves still touch the ground), (ii) adults before flowering and (iii) flowering and fruiting adults. For the randomly selected representative stands (covering all communes and altitude ranges of the adult categories in each commune), we measured the diameter at breast height and height as well as the geographical coordinates and other site characteristics (types of crops, presence of a water source, soil type, etc.) For dendrometric data, we measured 337 individuals from 52 stands. The height and diameter of *Raphia ruwenzorica* stands were measured using standardized dendrometric techniques. The diameter at breast height (DBH) was measured for all adult individuals using a tree caliper. Tree height was measured using a graduated telescopic pole for shorter individuals (typically those under 5 m) and a clinometer for the tallest specimens. For clinometer measurements, the distance from the tree was recorded with a tape measure, and height was calculated using standard trigonometric principles. Herbarium samples of *Raphia ruwenzorica* were collected and dried in the oven at 40°C for five days and placed in the freezer for three days, mounted in folders then labeled to finally be preserved. A total of 22 herbarium samples were collected and deposited in the Herbarium at the University of Burundi (BJA) and the doubles at Meise Botanic Garden Harbarium (BR).

#### 2.2.2 Socio‐Economic Data

##### 2.2.2.1 Sample Size Determination

The sample size on the socio‐economic importance was determined using the formula as described by Mugenda and Mugenda (2003). Using the 2008 Burundi Central Bureau of Census data on the population of Bujumbura rural communes and communes of Rumonge province (Table 1), the target population was 429,171 respondents. The sample size was determined as follows in Equation (1):

(1) $$n=\frac{N}{1+N{\left(e\right)}^{2}}$$

where *n* = the desired sample size (if the target population is greater than 10,000); *N* = population size; *e* = accepted level of error taking alpha as (*e* = 5% or 0.05).

$$n=\frac{429,171}{1+429,171{\left(0.05\right)}^{2}}=429,171/1073.93$$

*n* = 399.

**TABLE 1:** Sample size distribution.

	Population size	Sample size
*Bujumbura Province*		
Kanyosha	78,823	73
Nyabiraba	50,554	47
Kabezi	49,079	46
Mutambu	43,763	41
*Rumonge Province*		
Bunyengero	58,670	55
Burambi	57,167	53
Bugarama	30,482	28
Muhata	60,633	56
Total	429,171	399

*Source:* Burundi Central Bureau of Census (2008).

##### 2.2.2.2 Sample Distribution

The 399 sample households were proportionately allocated between the sample communes based on their effective total population as shown in Table 1. Out of 399 questionnaires, a sample of 181 questionnaires were used in analysis while 218 questionnaires were rejected due to 218 questionnaires were rejected during data cleaning due to incompleteness or containing significant internal inconsistencies. Respondents were primarily households located near Raphia stands, including users, harvesters, and artisans involved in the species' value chain. The selection criterion was being a resident over 30 years of age and presumed to have longer‐term knowledge of the resource.

##### 2.2.2.3 Data Collection

Data on socio‐economic importance were collected through individual semi‐structured surveys of households from the local community. The interviews were based on people aged over 30 years and residents of communes in the study. The interviews focused mainly on the knowledge of the population on the socio‐economic importance of *R. ruwenzorica* , uses of the species, value of products and its conservation concern, its habitat and localities where the species has already disappeared. The economic value of *R. ruwenzorica* products was obtained through the direct market valuation method, that is, use value by documenting the diverse uses and calculating the relative frequency of citation (RFC) for each use. This was achieved by interrogating the respondents on the value of products and frequency of production. For annual product production, respondents were asked to estimate the annual production quantity and the local market price for each product. This was estimated and annual market value calculated based on the local market before conversion to US dollars as shown below in Equation (2):

(2) $$\begin{array}{c} \text{Annual Value}\;\left(\mathrm{USD}\right)=\left(\text{Quantity produced}\;\mathrm{per}\;\text{year}\right)\\ \;\times \left(\text{Price}\;\mathrm{per}\;\text{unit in}\;\mathrm{USD}\right) \end{array}$$

**FIGURE 1:**
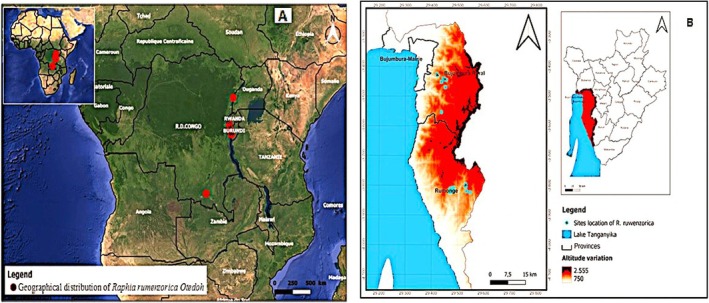
Geographical distribution of *Raphia ruwenzorica* in (A) global and (B) the South‐West of Burundi.

**FIGURE 2:**
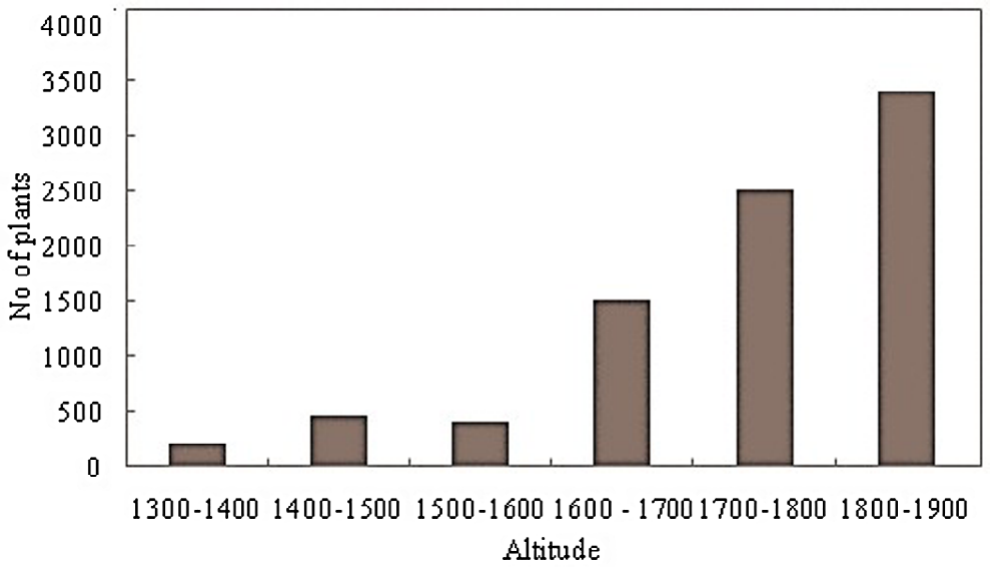
Variation in the number of *R. ruwenzorica* stands as a function of altitude in South‐West of Burundi.

The distribution map was produced using QGIS 2.18. The geographical coordinates (latitude/longitude) of all recorded *R. ruwenzorica* stands (*n* = 8172) collected during field transects were saved in a CSV file and imported into the software. These point data were overlaid on a base map of Burundi and the study area communes to generate the spatial distribution maps presented in Figures 1 and 2.

### 2.3 Data Processing and Analysis

Data analysis was conducted using a combination of spatial, statistical, and descriptive methods to address the study's objectives concerning distribution, ecology, and socio‐economic importance. Geographical coordinates for all recorded *R. ruwenzorica* stands (*n* = 8172) were compiled in a CSV file. These point data were imported into QGIS software (version 2.18, QGIS Development Team) and overlaid on base layers of Burundi and the study communes to produce the species distribution maps (Figures 1 and 2). The distribution of stands across the 100‐m altitudinal gradient was visualized using a graph. In the same way, the global distribution map of the species was produced using the herbarium sample coordinates from the field data and previous herbarium records from Meise Botanic Garden (BR). As suggested by Bamps (1982), we assigned locations for some herbarium samples missing coordinates. The scientific names of the species were checked in: http://www.ville‐ge/musinfo/bd/cjb/africana/, accessed June 2021. Descriptive statistics (mean, standard deviation) were calculated for the dendrometric variables (diameter at breast height—DBH, and height) for each stand category (young, adult before flowering, adult flowering) and commune. To test for significant differences in mean height and DBH among the different communes, a one‐way Analysis of Variance (ANOVA) was performed. Statistical significance was accepted at *p* < 0.05. All statistical tests were performed using SPSS software (version 25, IBM Corp.). Data from the semi‐structured questionnaires (*n* = 181) were coded and analyzed quantitatively and qualitatively. The socio‐economic importance of different plant parts was assessed using the Relative Frequency of Citation (RFC). The annual economic value of products derived from *R. ruwenzorica* was calculated using the direct market valuation method.

## 3 Results

### 3.1 Ecological Distribution of *Raphia Ruwenzorica*

The results obtained proved that the lowest altitude occupied by *Raphia ruwenzorica* is 1314 m located on Murara hill in Burambi commune while the highest is located at 1900 m altitude on Bubanza hill in Mutambu (Figures 1 and 2).

The distribution of the species in study area revealed its preference range between 1600 and 1900 m a.s.l. (Figure 2).

**FIGURE 3:**
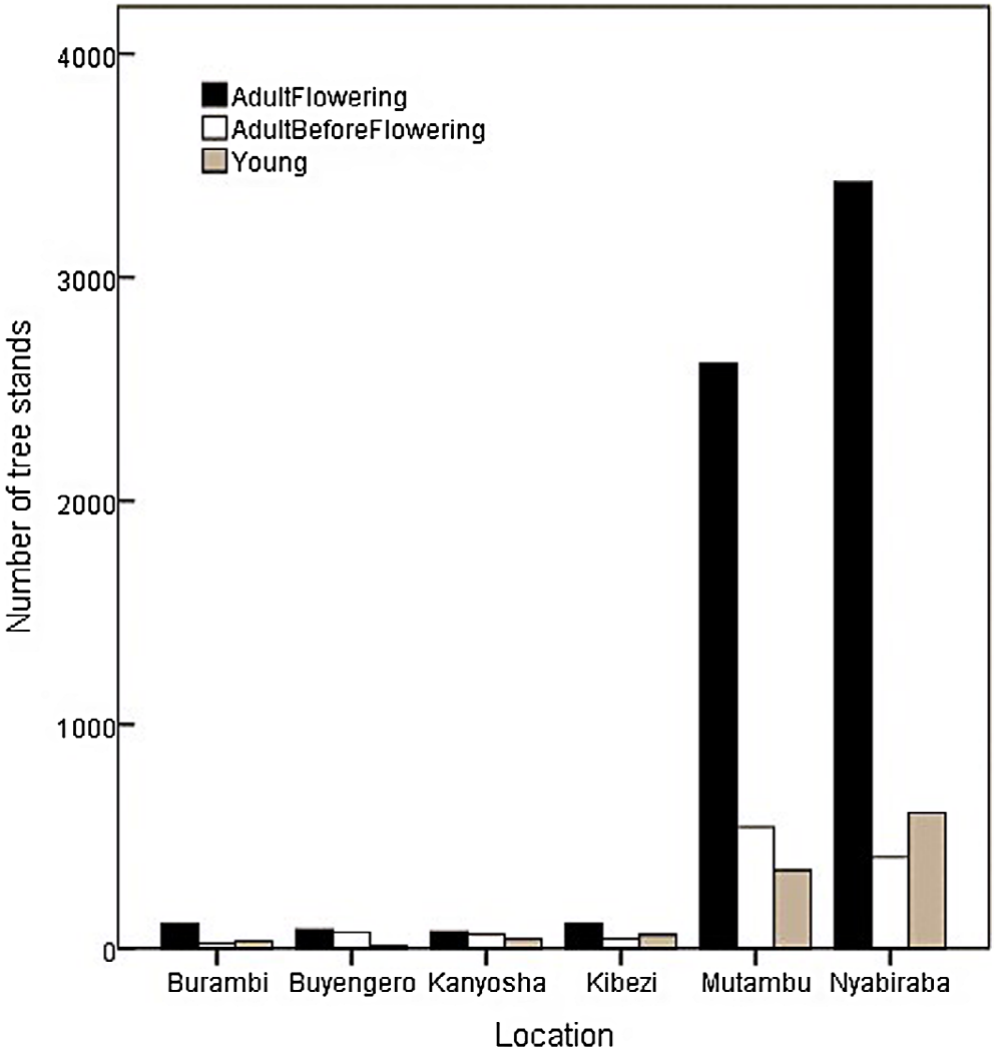
Number of *Raphia ruwenzoric*a stands recorded in the South‐West of Burundi.

A total of 8172 stands (distinct groups counted) of *Raphia ruwenzorica* were recorded in the study area (Figure 3). This includes 1181 (i.e., 14.45%) adult flowering stands, 5918 (i.e., 72.42%) adult before flowering and 1073 (i.e., 13.13%) young stands were recorded in the study area. Bujumbura province has the highest number of stands compared to Rumonge province with 7828 *Raphia ruwenzorica* stands iclunding14.09% of adult flowering stands, 72.58% of adult before flowering and 13.32% of young stands. Here, Nyabiraba and Mutambu communes contain more *Raphia ruwenzorica* stands (Figure 3). Rumonge province has a total of 344 stands that was composed of 22.67% of adult flowing stands, 68.60% of adult before flowering and 8.72% of young stands. This study revealed a very small number of young stands and adult flowering compared to the adult before flowering stands. The species was not found in the communes of Muhuta and Bugarama (Rumonge province) during our field surveys, and therefore they are not represented in Figure 3.

### 3.2 Height and Diameter of Identified *Raphia ruwenzorica*

The height and diameter of the flowering adult stands of the species varied from 4.66 ± 1.04 to 8.96 ± 2.2 m and 0.42 ± 007 m to 0.53 ± 0.12 m, respectively, while those of the adult stands before flowering varied from 3.48 ± 0.85 to 6.74 ± 1.35 m and 0.50 ± 0.09 to 0.59 ± 0.04, respectively (Table 2).

**TABLE 2:** Estimated height (m) and diameter (m) of *Raphia ruwenzorica* stands in different sites of the South‐West of Burundi.

Commune	Sample (*n*)	Height of flowering stands (m)	Height of adult before flowering (m)	Diameter of flowering stands (m)	Diameter of adult before flowering (m)
Kanyosha	45	4.66 ± 1.04	3.48 ± 0.85	0.45 ± 0.15	0.50 ± 0.11
Nyabiraba	72	5.64 ± 1.3	4.10 ± 1.17	0.42 ± 007	0.50 ± 0.09
Kabezi	53	7.8 ± 1.74	5.40 ± 1.21	0.50 ± 0.09	0.56 ± 0.08
Mutambu	75	8.96 ± 2.2	6.74 ± 1.35	0.45 ± 0.05	0.55 ± 0.07
Buyengero	45	7.2 ± 0.79	5.26 ± 0.8	0.47 ± 0.03	0.59 ± 0.04
Burambi	47	7.14 ± 2.07	4.25 ± 1.63	0.53 ± 0.12	0.60 ± 0.18

Abbreviations: m, meters; *n*, number of individuals measured.

### 3.3 Dendrometric Variation

One‐way Analysis of Variance (ANOVA) was conducted to test for significant differences in dendrometric characteristics across communes. There was a highly significant difference in all measured variables (Table 3). Height of flowering stands showed the most pronounced variation (*F*(5, 435) = 85.3, *p* < 0.001), with Mutambu containing significantly taller individuals (8.96 ± 2.2 m) compared to other communes. Height of adult before flowering stands also varied significantly (*F*(5, 435) = 72.1, *p* < 0.001), following a similar spatial pattern. Diameter measurements showed more moderate but still significant variation among communes for both flowering (*F*(5, 435) = 8.2, *p* < 0.001) and non‐flowering adults (*F*(5, 435) = 12.6, *p* < 0.001). Post hoc Tukey's HSD tests confirmed the specific group differences detailed in Table 3. The substantial variation in height across communes suggests strong environmental or management influences on growth patterns.

**TABLE 3:** Statistical analysis of dendrometric variation in *Raphia ruwenzorica* across communes.

Variable	*F*	*p*	Significant group differences (Tukey's HSD)
Height of flowering stands	*F*(5, 435) = 85.3	< 0.001	Mutambu > Kabezi > Buyengero ≈ Burambi > Nyabiraba > Kanyosha
Height of adult before flowering	*F*(5, 435) = 72.1	< 0.001	Mutambu > Kabezi > Buyengero > Nyabiraba > Burambi > Kanyosha
Diameter of flowering stands	*F*(5, 435) = 8.2	< 0.001	Burambi ≈ Kabezi > other communes
Diameter of adult before flowering	*F*(5, 435) = 12.6	< 0.001	Burambi > Buyengero ≈ Kabezi > Mutambu ≈ Nyabiraba ≈ Kanyosha

### 3.4 The Socio‐Economic Importance of Raphia Ruwenzorica

**FIGURE 4:**
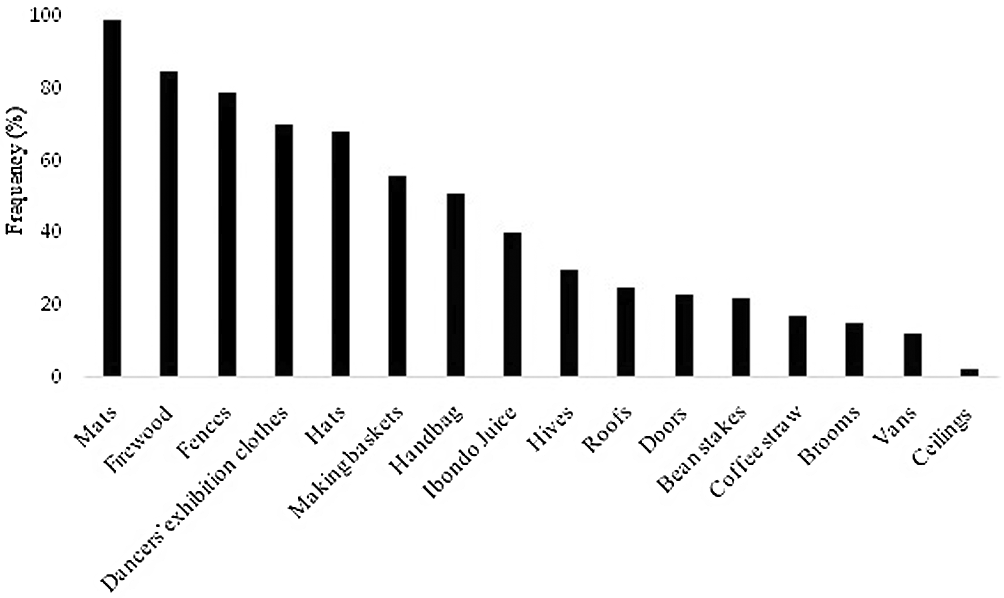
Different uses of *Raphia ruwenzorica* in the South‐West of Burundi.

The results obtained on the importance of *Raphia ruwenzorica* showed that almost all parts of the species are usable. For instance (Figure 4), the petiole of the leaves of *Raphia ruwenzorica* is used for various purposes: firewood (85.08%), construction of stables and fences (79.01%), manufacturing of baskets (56.91%), beehives (29.83%), doors (23.20%), vans (12.71%), bean staking (6.63%), ceilings (2.21%), shelves (1.1%), cabinets (1.1%), and toys (0.5%). The entire leaf of the species is used as roofs (kitchens/barns) (23.76%) and for the mulching of the coffee tree (3.31%). Its veins are used in the manufacture of brooms (2.76%) while its fibers are used in various activities. They are used as rope in the manufacture of mats (100%), exhibition clothes of Intore and Umuyebe dancers (70.17%), hats (68.51%), handbags (53.59%) as well as the belt to hold the umbilical cord of a newborn (0.55%).

The different uses of the stem or the trunk of *Raphia ruwenzorica* were mentioned: firewood (53.59%), manufacture of hives (95.03%) used in beekeeping, juice called “ibondo” drawn from the trunk (41.99%), which can also be used to alcoholize banana juice to produce banana wine, and this is mainly known in Buyengero and Burambi communes of Rumonge province.

The usefulness of seeds and roots of *Raphia ruwenzorica* by the population of the study area is less reported. The *Raphia* seeds were only reported for the production of seedlings while the roots were recognized for soil stabilization and reduction of erosion as well as the collapse of river banks.

The annual economic value of the products made from *Raphia ruwenzorica* was evaluated (Table 3). Individuals who own some *Raphia ruwenzorica* stands provided the estimation of economic value that ranges from $1.44 to $14.3 US dollars per year (Table 4). We considered the price of a leaf of *Raphia ruwenzorica*, where it ranked first economically among existing native stands in the study area.

**TABLE 4:** Economic value of some products made from *Raphia ruwenzorica*.

Types of products	No of products and stand part	Price of an item in US dollars	Annual value/stem in US dollars
Mats	2	4.8–9.6	28.8–57.7
Hats	1–2	0.9–2.8	2.88–17.3
Handbags	1	2.4–5.8	7.2–17.3
Trivet	1–5	16.8–19.2	16.8–19.2/2 stem
Coaster	5	0.7–1.4	10.8–21.6
Games	3	0.96–2.4	No annual

### 3.5 Conservation Status and Decreasing Use of Species

**FIGURE 5:**
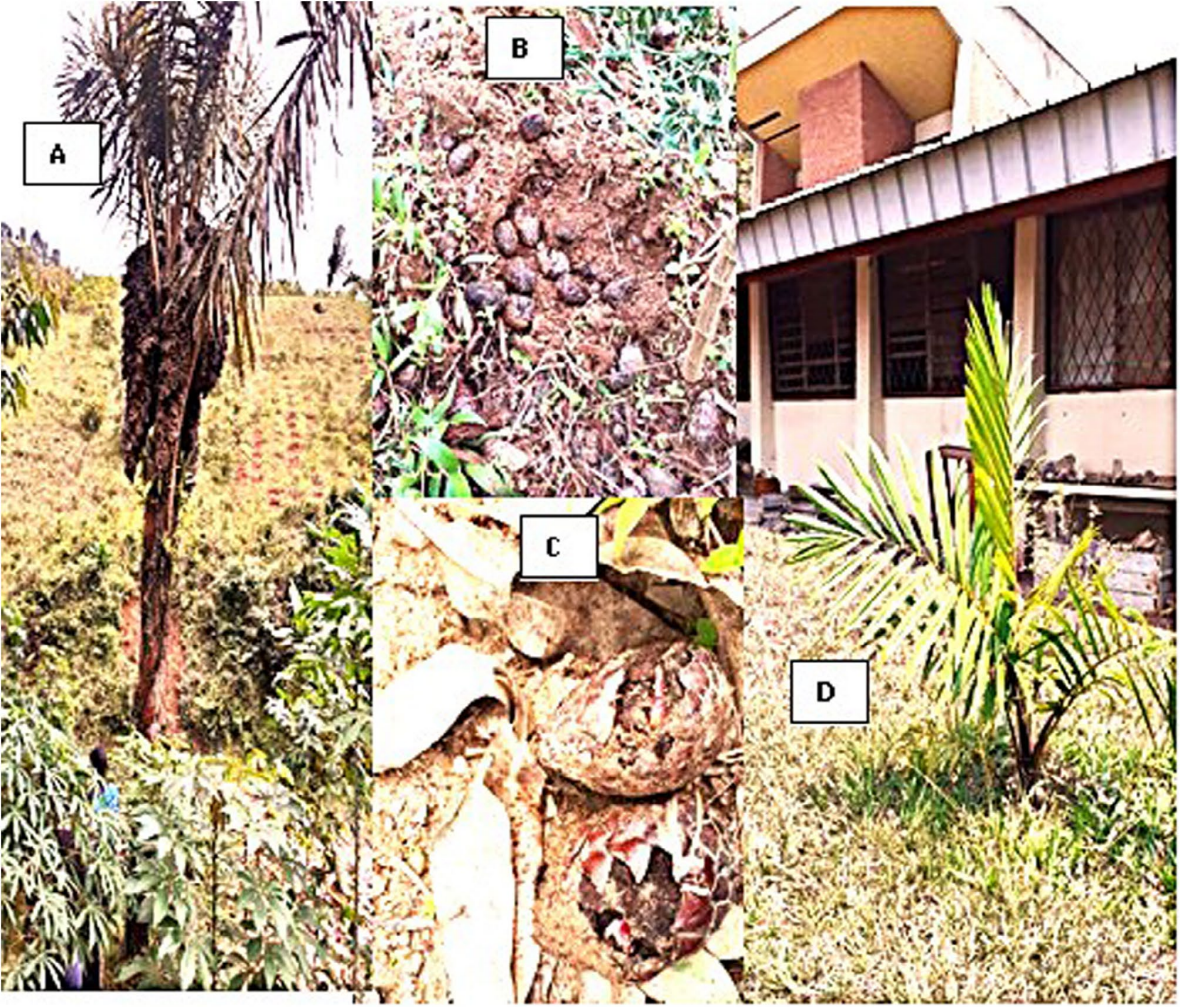
*Raphia ruwenzorica* flowering stand (A) with seeds germinating in humid soil (B, C) which can grow to a seedling (D) with many purposes environmental (D). Photo taken by the author: Prof. Jacques Nkengurutse.

In the study area, 63.54% of respondents affirmed that the number of *Raphia ruwenzorica* stands is declining (Table 5). They reported that major causes of the decline of this species were in particular the non‐replacement of aging stands (48.70%), the extension of cultivable fields (30.43%), and landslide along river banks (26.09%). The reduction in the use of resources from the species was reported by respondents due to the emergence of other alternative sources of raw materials for similar products. This is also supported by the scarcity of other materials used in the manufacturing of mats (15.65%), such as materials obtained from *Typha domingensis* and *Cyperus latifolius* plant species, as well as the low economic yield (14.78%), the loss of fiber value (9.57%), the use of mattresses instead of mats (8.70%), and the decrease in soil fertility (0.87%). These factors lead to a lack of motivation of the population to maintain and preserve this species. Fortunately, the population affirmed that they master the seedling production techniques from seed germination to sapling and adult stands (Figure 5).

**TABLE 5:** Population perception of the conservation status of *R. ruwenzorica* .

Conservation status (% of respondents)	Reason (% of respondents)
Progression (36.46%)	*R. ruwenzorica* planting (13.23%)
	Natural regeneration (83.82%) No‐reason (7.13%)
Regression (63.543%)	Landslide effects (26.09%)
	Agriculture expansion (30.43%)
	Decrease of population interest (25.66%)
	No replacement (48.70%)

## 4 Discussion

### 4.1 Spatial Distribution of Raphia Ruwenzorica From South‐West of Burundi

From our findings, *Raphia ruwenzorica* is a species that dominates riparian sites of rivers and streams. These data are consistent with findings by Mogue Kamga et al. (2020) who showed that most species of the genus *Raphia* are well adapted to humid habitats in Cameroon and are generally found in swamps, near watercourses and gallery forests. Our results revealed that *R. ruwenzorica* occurs between 1300 and 1900 m a.s.l. Previous studies have reported that *R. ruwenzorica* occurs between 800 and 1500 m (Helmstetter et al. 2020) and between 800 and 2000 m (Mogue Kamga et al. 2020) in the Albertine Rift region of eastern Democratic Republic of Congo, Cameroon and Nigeria. This spatial distribution indicates the species' ability to adapt to varying environments and a wide range of altitude. In the current study, the species preferred an altitude ranging between 1600 and 1900 m, as a large number of stands was found in this altitude range. Although we generated Figure 1 map of distribution, counting the exact number of individual stands was very difficult and time consuming. Therefore, the current study does not produce an accurate comprehensive inventory of *R. ruwenzorica* plantation. Additionally, our results represent a population count and point distribution, not an area‐based coverage. To overcome these limitations of ground‐based inventories in complex riparian terrain, future research should employ advanced remote sensing tools (Culman et al. 2020; Hajjaji et al. 2023). High‐resolution satellite imagery and drone‐based surveys are particularly promising for efficiently delineating individual tree crowns or stands and generating precise distribution maps, as demonstrated in studies of other palm species and non‐timber forest products (Culman et al. 2020).

Our findings implied that the species is likely to vary between low and high altitude. The strong interactions between biotic and abiotic components have been shown to regulate species density based on elevation (Parmesan and Yohe 2003; Ouattara et al. 2023). It is possible that our findings could also be explained by differences in the human population density and utilization of the species. The high occurrence of the species in high altitude may be due to the relatively low land use in this region. A more detailed study is relevant to reveal the determinants of the occurrence of this species in Burundi and in its geographical area of distribution as presented in Figure 1B.

### 4.2 Socio‐Economic Importance of Raphia Ruwenzorica From South‐West of Burundi

*Raphia* palm is one of the most economically useful palms (Obahiagbon 2009). Previous studies carried out in Benin, Madagascar, Côte d'Ivoire, and Cameroon show that *Raphia* species are widely used for crafting numerous artifacts and general purposes (Donou Hounsode et al. 2016; Byg and Balslev 2001; Sibirina et al. 2014; Mphoweh et al. 2015). Mogue Kamga et al. (2020) reported nearly one hundred different uses across the genus. This multipurpose utility is consistent with findings across the *Raphia* genus, which is renowned for its wide array of applications (Mogue Kamga et al. 2020). In this study, the most cited uses of the species were based on products made from fibers. *R. ruwenzorica* fibers were basically important for manufacture of baskets and exhibition clothes for *Intore* and *Umuyebe* traditional dancers, as well as making hats, handbags, and mats. Similar findings have been reported for the cultural importance of other palms by Mogue Kamga et al. (2020). Apart from the fiber uses, the community mainly used *R. ruwenzorica* for manufacturing beehives using its trunk, construction of fences, and the petioles were used for firewood. The values of products ranged from $1.44 to $14.3 US dollars per year. This represents annual cash income generated per stem from specific crafted products (e.g., mats, hats), not the total value of all services provided by a stand. Our partial valuation, however low, represents supplementary artisanal income within a local, informal market economy, which is common for Non‐Timber Forest Products (NTFPs) (Ouattara et al. 2023).

The growth habit of *R. ruwenzorica* is a cylindrical with medium height trunk. The trunk of this palm is made of vascular bundles held together with connective tissue. Towards the periphery, where the leaf bases are embedded, the tissue tends to become more lignified and tough which is similar to other palm trees species such as the date palm (Barreveld 1993). The height varied between adult flowering and before flowering where a maximum height of 8.96 ± 2.2 m and 6.74 ± 1.35 m were recorded, respectively. The trunks were slender with slightly similar diameter at breast height ranging from 0.42 ± 007 to 0.60 ± 0.18 m for the tree stands. Utlization of the tree involved cutting old leaves at the base of the leaf stem. This leads to the trunk becoming much smaller in diameter as described by Al‐Suhaibani et al. (1988) in other palm trees. These height and diameter are also relevant in terms of economic use of the species. The bigger and taller the trunk, that is, with large diameter, the more material is available for fabrication of items (e.g., beehives or construction material) and the more earnings for the communities.

*Raphia* resources constitute the pre‐colonial currency. The terms expressing money such as *makuta* and *mbongo*, commonly used in the Congo Basin from Swahili, Kikongo, and Lingala, originate in the types of clothing made from *Raphia* fibers (Mogue Kamga et al. 2020). In Burundi, *Raphia ruwenzorica* fibers (*umuhivu*, in Kirundi) are compared to the precious womb of a mother (“agahivu ko munda gacika ntibunge”, in Kirundi). Some respondents reported the use of *Raphia ruwenzorica* fibers to hold the umbilical cord of newborns. However, this practice seems to be disappearing as few respondents reported it. The same applies to wine made from the *Raphia ruwenzorica*'s juice, *Ibondo*. Currently *Ibondo* means for many the wine from oil palm ( *Elaeis guineensis* ) juice, but this is true also for the vernacular name of *R. ruwenzorica* . Since the oil palm is not indigenous to Burundi, *Ibondo* as wine would take its name from *Raphia ruwenzorica*. Therefore, if few people report wine made from this species, this seems to indicate an important use that is being abandoned, probably to use alternative drinks. Furthermore, we hypothesize a competitive exclusion in low‐altitude areas suitable for oil palm, and a preservation effect for *R. ruwenzorica* in higher altitudes where oil palm is less profitable. We also note that the species is primarily spontaneous but tolerated or encouraged in certain settings. We believe that some uses of the species are being abandoned due to the availability of alternative materials for crafting products and hence the loss of traditional related knowledge.

### 4.3 Conservation of Raphia Ruwenzorica From South‐West of Burundi

This spatial restriction within the for mid‐altitudes is compounded by a population structure dominated by non‐flowering adults, indicating limited regeneration that threatens long‐term sustainability. The significant variation in tree height among communes, as confirmed by ANOVA, further suggests that local environmental conditions or management practices directly influence growth patterns. Despite these threats, the species remains a vital socio‐economic resource, with its fibers integral to cultural artifacts like traditional dance costumes as well as contributing modest but important artisanal income. Consequently, the conservation of *R. ruwenzorica* requires strategies that address both environmental constraints and economic incentives to ensure this multi‐purpose palm remains a component of the agricultural landscape.

We hypothesized that *R. ruwenzorica* population is decreasing because of the increasing human population in South‐West of Burundi. This assumption seems not to be consistent. In fact, the data from General Population Census of 2008 show that the communes where the species is well represented (Nyabiraba and Mutambu) had a relatively high density of human population of the study area communes (ISTEEBU 2008). We believe that the current *R. ruwenzorica* distribution and abundance can be explained by the palm oil culture in the communes. In fact, *R. ruwenzorica* has a geographical range (between 800 and 2000 m a.s.l.) comprising that of the palm oil (Mogue Kamga et al. 2020; Murugesan et al. 2017). As mentioned above, the South‐West population of Burundi is very enthusiastic about palm oil culture. We assume that this may have led to the abrupt decrease of the *R. ruwenzorica* population. Furthermore, because of the increase in population and the increased demand for agricultural land, we believe that the adoption of *R. ruwenzorica* species will have to be done in cohabitation with the oil palm culture. As *R. ruwenzorica* is listed as Data Deficient (Cosiaux, Gardiner, and Couvreur 2018) due to lack of sufficient data to allow IUCN Red‐List assessment (Cosiaux, Gardiner, Stauffer, et al. 2018), we believe that the results of the present study and its consecutive herbarium sample collection constitute a great contribution to the IUCN Red‐List assessment.

## 5 Conclusion

The objective of this study was to contribute to the identification of the geographical distribution and socio‐economic importance of *Raphia ruwenzorica* in Burundi. The results show that *Raphia ruwenzoric*a is present in several communes such as Kabezi, Kanyosha, Nyabiraba, and Mutambu of Bujumbura province and in Buyengero and Burambi communes of Rumonge province. The species occurs between 1300 and 1900 m and is more abundant between 1600 and 1900 m. The inventory recorded 8172 stands of *Raphia ruwenzorica* with 1181 adult flowering, 5918 adults before flowering, and 1073 young stands. *R. ruwenzorica* is important to the population, who utilizes all parts of the plant to craft different products. Unfortunately, the population of this species is declining due to agricultural expansion, no replacement, and decrease of population interest. Consequently, the traditional heritage is also being lost. For effective conservation, we recommend integrating *R. ruwenzorica* into on‐farm agroforestry systems, promoting community‐based nurseries to enhance regeneration, and raising awareness of its cultural and economic value to incentivize its sustainable management. Future research should employ a deliberately stratified sampling design to investigate the important roles of gender, age, and spatial patterns in the use and knowledge of this valuable species.

## Funding

The study was supported by the CEBios Program of the Royal Belgian Institute of Natural Sciences through the “Programme de recherche, échange d'information, sensibilisation et conservation de la biodiversité au Burundi” implemented by the Office Burundais pour la Protection de l'Environnement (OBPE) and the National Natural Science Foundation of China (Grant No. 31961143023).

## Conflicts of Interest

The authors declare no conflicts of interest.

## Data Availability

The data that support the findings of this study are available at the Université du Burundi institutional repository (DSpace platform) at https://www.ub.edu.bi/Publication/syllabus_article/270.
